# Supplementary material for: An astrocytic signaling loop for frequency-dependent control of dendritic integration and spatial learning
Source: Nat Commun. 2022 Dec 24;13:7932. doi: 10.1038/s41467-022-35620-8 (PMC9789958; doi:10.1038/s41467-022-35620-8)
Supplement: Supplementary file 1 — Supplementary Material [file 41467_2022_35620_MOESM1_ESM.pdf]

Supplementary figure 1

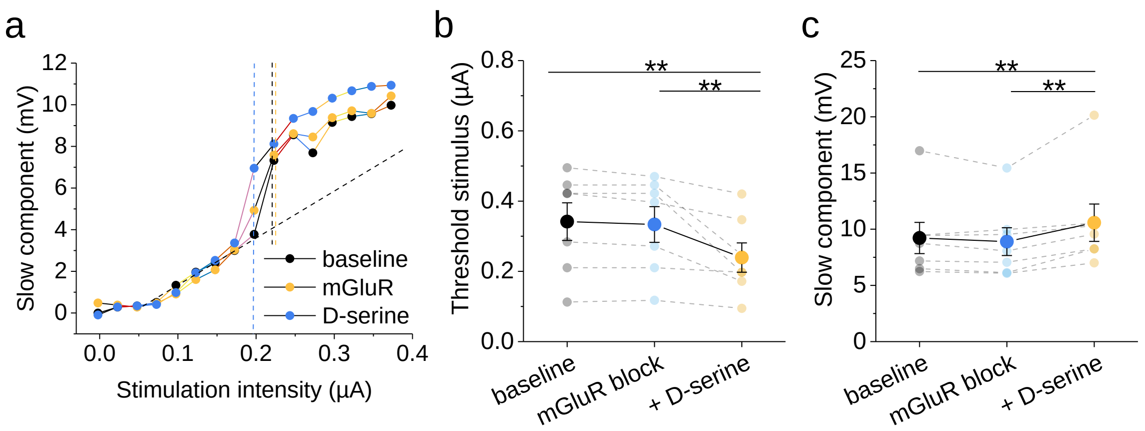

Supplementary figure 1: Activity of metabotropic glutamate receptors (mGluRs) does not control dendritic spikes evoked by iontophoretic glutamate application.

a) Sample experiment probing dendritic spiking under baseline conditions (black), in the presence of the mGluR inhibitors LY341495 (50 µM) and MPEP (10 µM) (yellow), which at these concentrations should block all relevant mGluRs, and after additional wash-in of D-serine (10 µM, blue).

b) Threshold stimulus of evoking a dendritic spike (0.34 ± 0.05 µA vs. 0.33 ± 0.05 µA vs. 0.24 ± 0.04 µA from left to right, n = 7, F(1.03,6.19) = 9.50, p = 0.020, one-way repeated-measures ANOVA; post-hoc Fisher LSD: baseline vs. mGluR t(12) = 0.31, p = 0.76, baseline vs. D-serine t(12) = 3.92, p = 0.0020, mGluR vs. D-serine t(12) = 3.61, p = 0.0036).

c) Amplitude of slow component of the dendritic spike (9.22 ± 1.39 mV vs. 8.89 ± 1.24 mV vs. 10.58 ± 1.66 mV from left to right, n = 7, F(1.08,6.47) = 10.59, p = 0.015 one-way repeated measures ANOVA; post-hoc Fisher LSD: baseline vs. mGluR t(12) = 0.83, p = 0.42, baseline vs. D-serine t(12) = 3.51, p = 0.0043, mGluR vs. D-serine t(12) = 4.33, p = 0.00097).

Data are expressed and displayed as mean ± s.e.m. Source data are provided as a Source Data file.

Supplementary figure 2

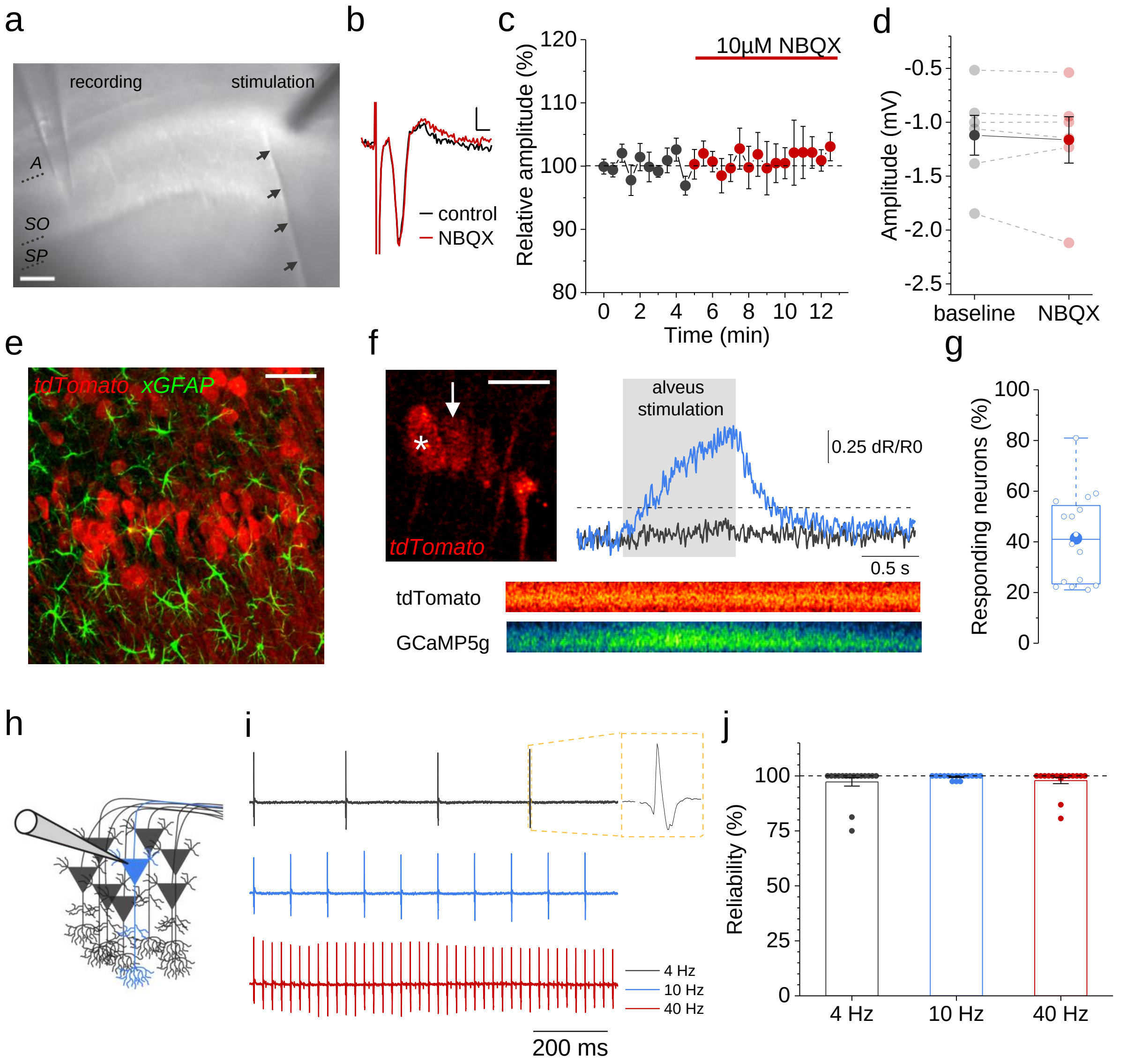

Supplementary figure 2: Experimental approach for retrogradely evoking CA1 pyramidal cell activity by alveus stimulation.

a) Placement of extracellular recording and stimulation electrodes in the CA1 pyramidal cell layer (SP) and alveus (A), respectively (SO, stratum oriens). Arrows indicate position of a cut that was made to isolate retrograde activation of CA1 pyramidal cells by stimulating their axons.

b) Example of a population spike evoked by stimulation in the alveus (scale bar, 0.1 mV, 1 ms).

c-d) The amplitude of the evoked population spike is stable over time (**c**) and independent of excitatory synaptic transmission (**d**, population spike amplitude, control -1.12 ± 0.18 mV, NBQX -1.16 ± 0.21 mV, n = 6, t(5) = 0.76, p = 0.48, two-sided paired Student's t-test).

e) Virally induced recombination in CA1 pyramidal cells using a CaMKII-cre AAV in a mouse line with cre-dependent expression of GCaMP5g and tdTomato [1]. Staining for GFAP and tdTomato revealed no recombination in astrocytes (confocal microscopy, scale bar 50 µm).

f) CA1 pyramidal cells expressing tdTomato in an acute slice (top left panel, scale bar 20 µm). Line scans through several somata were performed during stimulation of the alveus (examples in bottom panels, width of line scan 8.5 µm, length 2129 ms). Top right panel: representative filtered traces of the ratio of GCaMP5g and tdTomato (background-subtracted and normalized to baseline) are illustrated for a nonresponding (asterisk, grey trace) and a responding neuron (arrow, blue trace). Cells were considered responders if the ratio increased more than 25 % over baseline (dashed line).

g) On average, 41.3 ± 4.4 % of the cells in an acute brain slice (n = 16) responded to alveus stimulation.

h-i) Cartoon illustrating extracellular single unit recordings and examples for different frequencies of alveus stimulation.

j) The reliability of evoking single unit activity did not depend on the alveus stimulation frequency. When a pyramidal cell single unit response was identified blindly during 10 Hz stimulation of the alveus, several trains of stimuli were delivered at three frequencies (4, 10 and 40 Hz) and the probability of observing a unit spike was calculated. No significant differences were detected (4 Hz: 97.3 ± 1.9 %, 10 Hz: 99.5 ± 0.3 %, 40 Hz: 97.9 ± 1.4, n = 16 cells from 7 independent experiments, $F(1.01,15.13) = 1.55$, $p = 0.23$ one-way repeated-measures ANOVA).

Data are expressed and displayed as mean ± s.e.m. or in box plots. In the latter, the box indicates the 25th and 75th, the whiskers the 5th and 95th percentiles, the horizontal line in the box the median and the mean is represented by a filled circle. Source data are provided as a Source Data file.

Supplementary figure 3

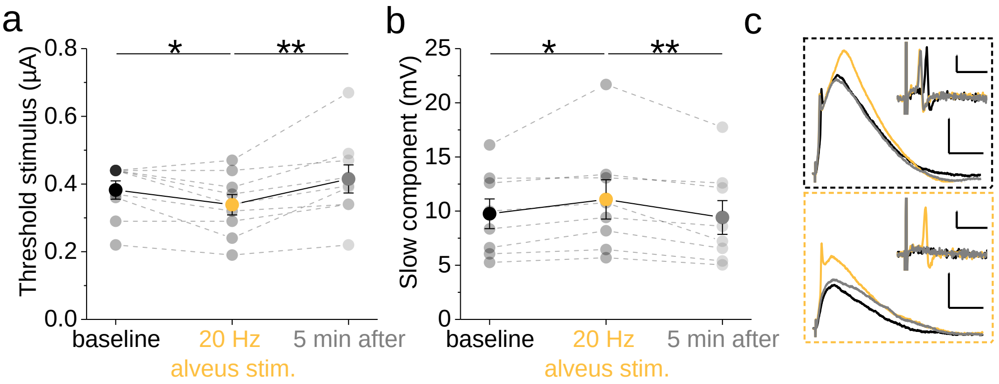

Supplementary figure 3: Changes of dendritic spikes induced by alveus stimulation are reversible. The threshold stimulus of dendritic spikes and their slow component were analyzed in baseline conditions, during 20 Hz alveus stimulus and 5 minutes after alveus stimulation.

a) Threshold stimulus during baseline, 20 Hz alveus stimulation, and five minutes later (baseline: 0.38 ± 0.03 µA, 20 Hz: 0.34 ± 0.03 µA, 5 min: 0.41 ± 0.04 µA, n = 9, $\chi^2(2) = 9.06$, p = 0.011 Friedman test; post-hoc two-sided Wilcoxon signed-rank tests: z = 2.12, p = 0.031 for baseline vs. 20 Hz, z = 1.26, p = 0.21 for baseline vs. 5 min, z = 2.61, p = 0.0039 for 20 Hz vs. 5 min).

b) Analysis of the slow component of dendritic spikes of the same recordings. n = 8 because somatic spikes prevented analysis of the slow component in one recording (baseline: 9.75 ± 1.37 mV, 20 Hz: 11.08 ± 1.81 mV, 5 min: 9.41 ± 1.56, n = 8, F(2,14) = 5.80, p = 0.015 one-way repeated measures ANOVA; post-hoc Fisher's LSD test, t(14) = 2.57, p = 0.022 for baseline vs. 20 Hz, t(14) = 0.66, p = 0.52 for baseline vs. 5 min, t(14) = 3.22, p = 0.0061 for 20 Hz vs. 5 min).

c) Sample traces for baseline (black), 20 Hz alveus stimulation (yellow) and 5 min after (grey). The upper panel (black border) shows traces for all three conditions recorded with the stimulus intensity that just evoked a dendritic spike under baseline conditions (threshold stimulus). Note that during alveus stimulation the slow component is increased. The lower panel (yellow border) shows traces for all three conditions recorded with the stimulus intensity that just evoked a dendritic spike during 20 Hz alveus stimulation. Because alveus stimulation reduces the threshold stimulus, no dendritic spikes occur at that simulation intensity under baseline conditions and 5 minutes after alveus stimulation. Insets display dV/dt with the same color coding. Scale bars 2 mV and 20 ms and in insets 2 mV/ms and 5 ms.

Data are expressed and displayed as mean ± s.e.m. Source data are provided as a Source Data file.

Supplementary figure 4

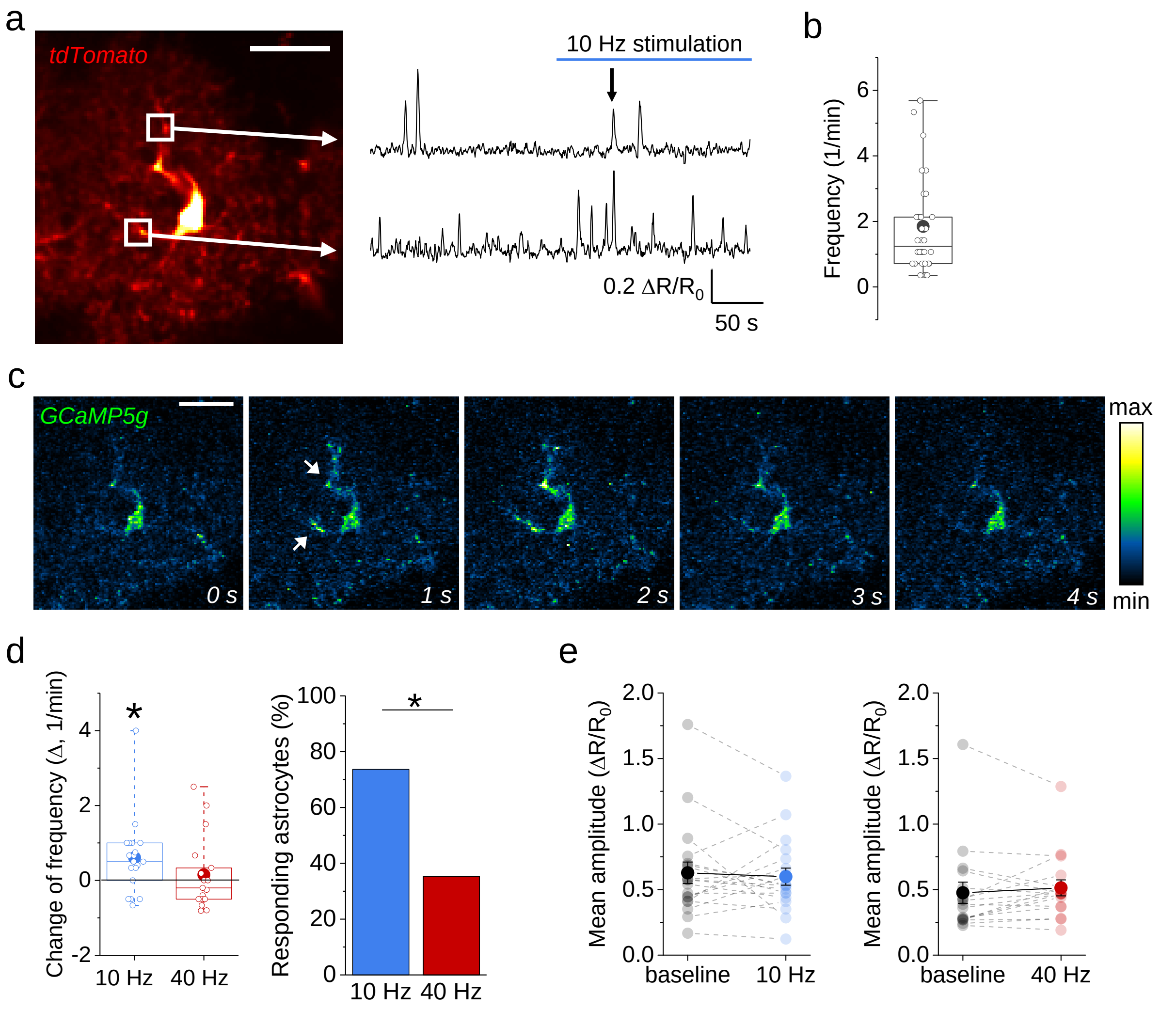

Supplementary figure 4: Frequency-dependent activation of astrocytes by alveus stimulation. Ca^{2+} transients were monitored in astrocytes expressing GCaMP5g and tdTomato. Additional analyses of the data presented in **Fig. 5a-c**. Also, see method sections for this analysis.

a) Example of an astrocyte expressing tdTomato (left panel, scale bar 20 µm) and GCaMP5g (illustrated in **c**). Right panel: examples of astrocytic Ca^{2+} transients from the two regions of interest (ROI) illustrated in the left panel. The fluorescence ratio (R) of GCaMP5g and tdTomato was calculated and its changes (ΔR) were normalized to its resting value (R_0). See Methods for further details.

b) Frequencies of Ca^{2+} transients during baseline recordings before alveus stimulation in active cells (1.85 ± 0.25 events per minute, n = 36 astrocytes).

c) Example of the change in GCaMP5g fluorescence over time (average intensity of three frames, scale bar 20 µm) for the Ca^{2+} transients marked with a black arrow in **a**. The two transients are marked with white arrows in **c**.

d) Change of Ca^{2+} transient frequency during alveus stimulation at 10 and 40 Hz (blue and red, respectively). Alveus stimulation at 10 but not 40 Hz significantly increased the frequency of Ca^{2+} transients (left panel; 10 Hz: 0.60 ± 0.23, n = 19, z = 2.45, p = 0.011; 40

Hz: 0.15 ± 0.24, n = 17, z = 0.17, p = 0.86; two-sided one-population Wilcoxon signed-rank tests). The percentage of astrocytes showing an increase in frequency was significantly higher with 10 Hz stimulation than with 40 Hz (right panel; 73.7 % vs. 35.3 %, p = 0.043, Fisher's exact test, n = 14 out of 19 cells for 10 Hz and 6 out of 17 cells for 40 Hz).

e) Alveus stimulation did not affect the amplitude of astrocytic Ca^{2+} transients (10 Hz, left panel: 0.63 ± 0.08 vs. 0.60 ± 0.07, n = 19, z = 0.46, p = 0.65; 40 Hz, right panel: 0.48 ± 0.08 vs. 0.51 ± 0.06, n = 17, z = 1.09, p = 0.28; two-sided Wilcoxon signed-rank tests). Individual data points represent the mean Ca^{2+} transient amplitude in an astrocyte.

Data are expressed and displayed as mean ± s.e.m. or in box plots. In the latter, the box indicates the 25th and 75th, the whiskers the 5th and 95th percentiles, the horizontal line in the box the median and the mean is represented by a filled circle. Source data are provided as a Source Data file.

Supplementary figure 5

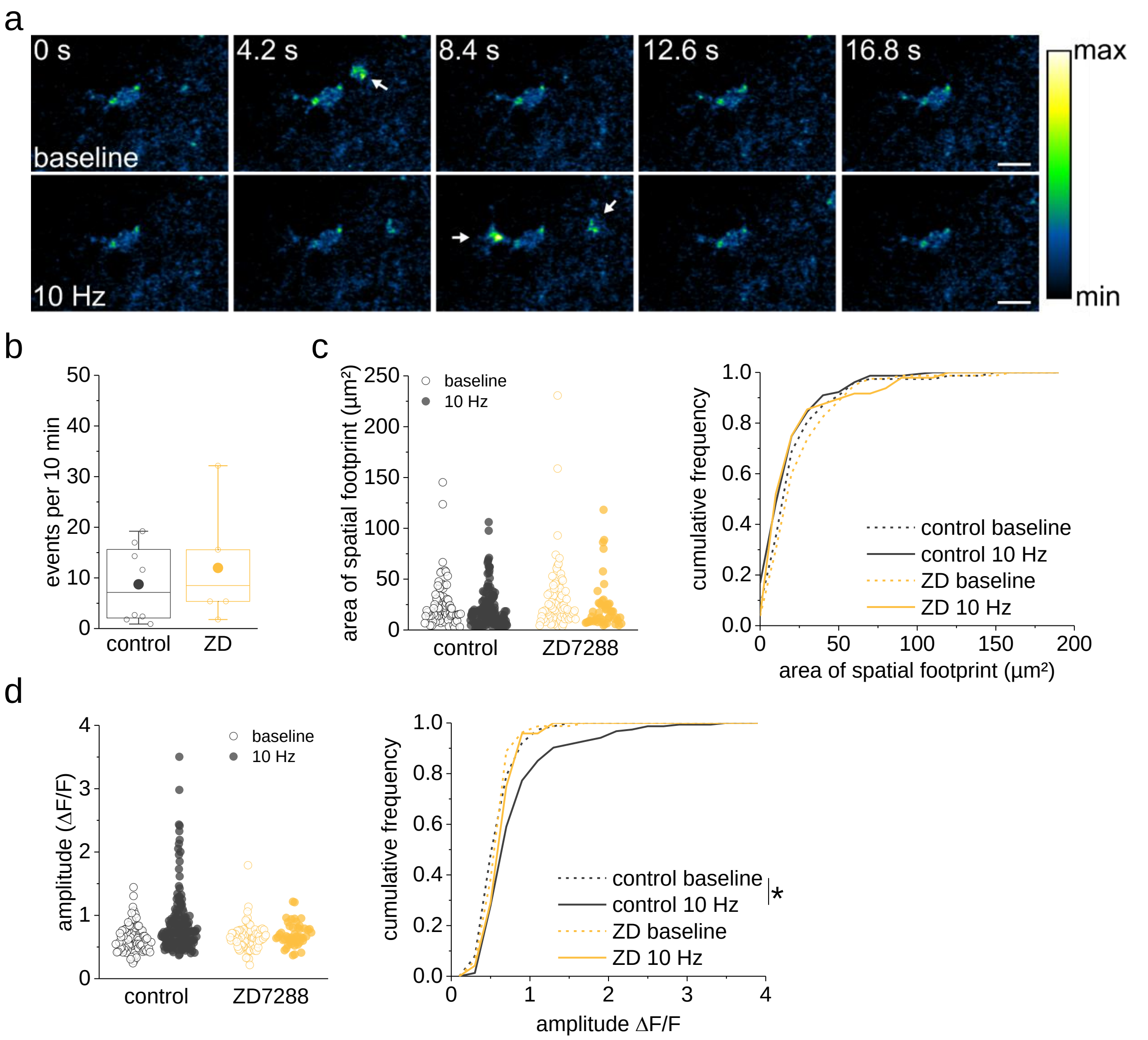

Supplementary figure 5: Activation of astrocytes by alveus stimulation is prevented by inhibition of HCN channels. Ca^{2+} events were monitored in astrocytes expressing GCaMP6f and detected using AQuA [2] (additional analyses of the data presented in **Fig. 5f-g**). Also see method section on this analysis.

a) Example of GCaMP6f fluorescence changes over time (average intensity of five frames) for the three events (marked by arrow) illustrated in Fig. 5f (scale bar 10 µm).

b) HCN channel inhibition by ZD7288 (10 µM) does not influence the basal Ca^{2+} event frequency (control vs. ZD7288: 8.73 ± 2.68 vs. 11.97 ± 4.51 events per 10 minutes, n = 8 and 6 cells, p = 0.64, z = 0.45; two-sided Mann-Whitney U-test).

c) Alveus stimulation at 10 Hz did not affect the area of detected events. Left panel shows the area of events detected by AQuA (control: baseline vs. 10 Hz, 25.44 ± 2.67 µm² vs. 20.06 ± 1.49 µm², n = 77 and 154 events from 8 cells; ZD7288: baseline vs. 10 Hz, 30.77 ± 3.62 vs. 22.90 ± 3.52 µm², n = 81 and 48 events from 6 cells). For further statistical analysis (right panel), the cumulative frequency of the observed event sizes was calculated (in 10 μm^2 bins) and compared between baseline and 10 Hz stimulation within each experimental group

(control and ZD) (control: n = 20 bins, p = 0.081, z = 1.26, two-sided Kolmogorov-Smirnov test; ZD7288: n = 20 bins, p = 0.83, z = 0.63, two-sided Kolmogorov-Smirnov test).

d) Alveus stimulation at 10 Hz increased the amplitude (maximum ΔF/F) of events detected by AQuA in control recordings but not in presence of the HCN inhibitor ZD7288 (control: baseline vs. 10 Hz, 0.64 ± 0.03 vs. 0.88 ± 0.04, n = 77 and 154 events from 8 cells; ZD7288: baseline vs. 10 Hz, 0.65 ± 0.02 vs. 0.70 ± 0.03, n = 81 and 48 events from 6 cells). For further statistical analysis (right panel), the cumulative frequency of the observed event amplitudes was calculated (0.2 ΔF/F bins) and compared within each experimental group (control and ZD) between baseline and 10 Hz stimulation (control: n = 20 bins, p = 0.012, z = 1.58, two-sided Kolmogorov-Smirnov test; ZD7288: n = 20 bins, p = 1.00, z = 0.32, two-sided Kolmogorov-Smirnov test).

The Kolmogorov-Smirnov test was chosen for **c** and **d** because data sets do not follow a normal distribution preventing further analysis with, for example, a two-way repeated measure ANOVA.

Data are expressed as mean ± s.e.m. in the legend and displayed in box plots. The box indicates the 25th and 75th, the whiskers the 5th and 95th percentiles, the horizontal line in the box the median and the mean is represented by a filled circle. Source data are provided as a Source Data file.

Supplementary figure 6

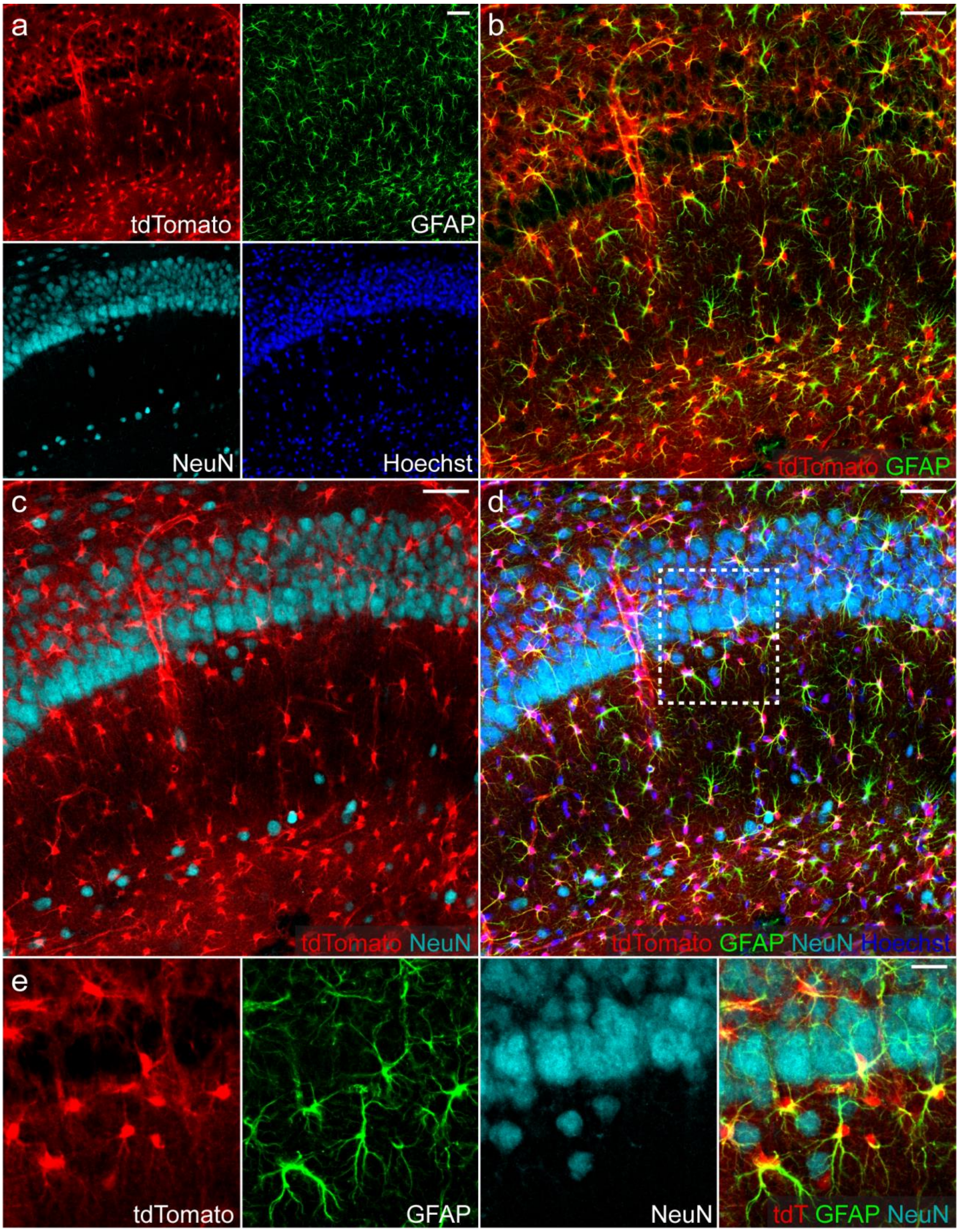

Supplementary figure 6: Characterization of astrocyte-specific recombination after tamoxifen-injection in GLASTcreERT2 mice in the CA1 region. GLASTcreERT2 [3] mice crossed with a flox-stop tdTomato reporter line [4] were injected with tamoxifen and their hippocampal CA1 region characterized by immunohistochemistry and confocal microscopy (n = 3 animals). Scale bars are 50 µm in **a-d** and 20 µm in **e**. In each animal, three sections (512 µm x 512 µm x 20 µm) were manually analyzed, and the results were averaged per mouse.

a) Examples of tdTomato expression (top left panel), astrocytic GFAP expression (top right panel), neuronal NeuN expression (bottom left panel) and DNA label Hoechst to visualize cell nuclei (bottom right panel)

b) The vast majority of tdTomato-expressing cells is positive for the astrocytic marker GFAP (overlay of tdTomato and GFAP). Of all tdTomato-expressing cells, 89.7 ± 1.4 % (186 ± 8 out of 207 ± 6 cells, n = 3 animals) were positive for GFAP. Of all GFAP-positive cells, 98.7 ± 0.8 % (186 ± 8 out of 188 ± 9 cells, n = 3 animals) expressed tdTomato.

c) Neuronal NeuN expression and tdTomato expression do not overlap (overlay of tdTomato and NeuN). Of all NeuN-positive cells, only 2.0 ± 1.8% (6 ± 5 out of 270 ± 30 cells, n = 3 animals) were also positive for tdTomato.

d) Overlay of tdTomato, GFAP, NeuN and Hoechst. A higher magnification of the delineated area is shown in **e**.

Data are expressed as mean ± s.e.m. in the legend. Source data are provided as a Source Data file.

Supplementary figure 7

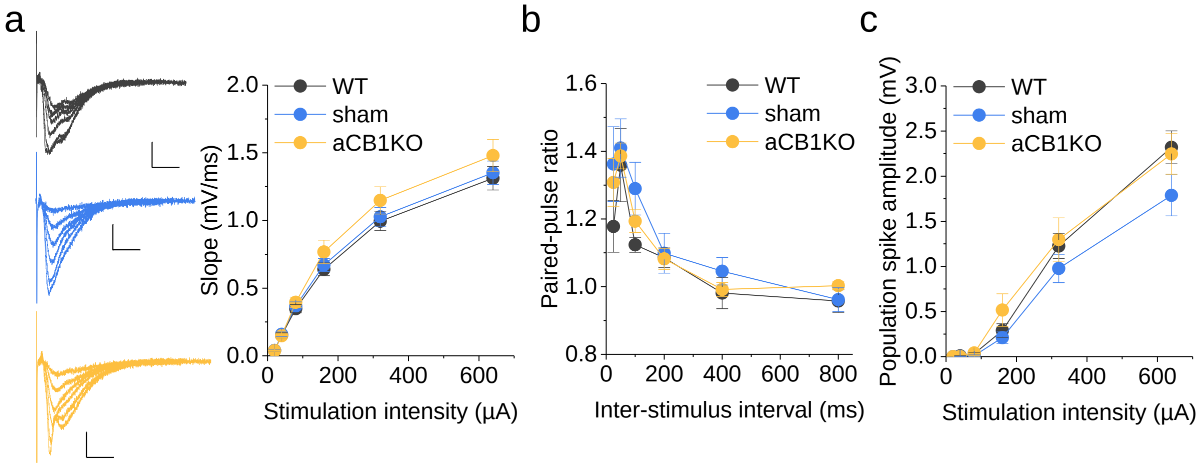

Supplementary figure 7: Basic properties of CA3-CA1 synaptic transmission are not altered by the absence of astrocytic CB1 receptors. Comparison of fEPSPs in CA1 stratum radiatum evoked by electrical stimulation of CA3-CA1 synapses in slices from wild-type animals (WT) and GLASTcreERT2 x CBR1 $^{fl/fl}$ x flox-stop tdTomato mice injected with tamoxifen (aCB1KO) or a control solution (sham).

a) Stimulus-response curve. Examples are shown on the left (top: WT, black; middle: sham, blue; bottom: aCB1KO, yellow; scale bars: 10 ms and 0.5 mV). Right panel: summary of experiments (WT: n = 49 from 19 animals; sham: n = 41 from 16 animals; aCB1KO: n = 46 from 17 animals; two-way repeated measures ANOVA, between groups, $F(2,133) = 0.80$, $p = 0.37$).

b) The paired-pulse ratio of fEPSP was recorded at several inter-stimulus intervals. No statistically significant differences were found between groups (WT: n = 7 from 4 animals, sham: n = 12 from 5 animals; aCB1KO: n = 14 from 6 animals; two-way repeated measures ANOVA, between groups, $F(2,30) = 0.77$, $p = 0.39$)

c) Population spikes were recorded from the CA1 pyramidal layer and evoked by stimulation of CA3-CA1 synaptic connections at various stimulation intensities. No statistically significant differences between groups (WT: n = 46 from 20 animals, sham: n = 32 from 17 animals; aCB1KO: n = 30 from 14 animals; two-way repeated measures ANOVA, between groups, $F(2,105) = 1.59$, $p = 0.21$).

Data are expressed and displayed as mean ± s.e.m. Source data are provided as a Source Data file.

Supplementary figure 8

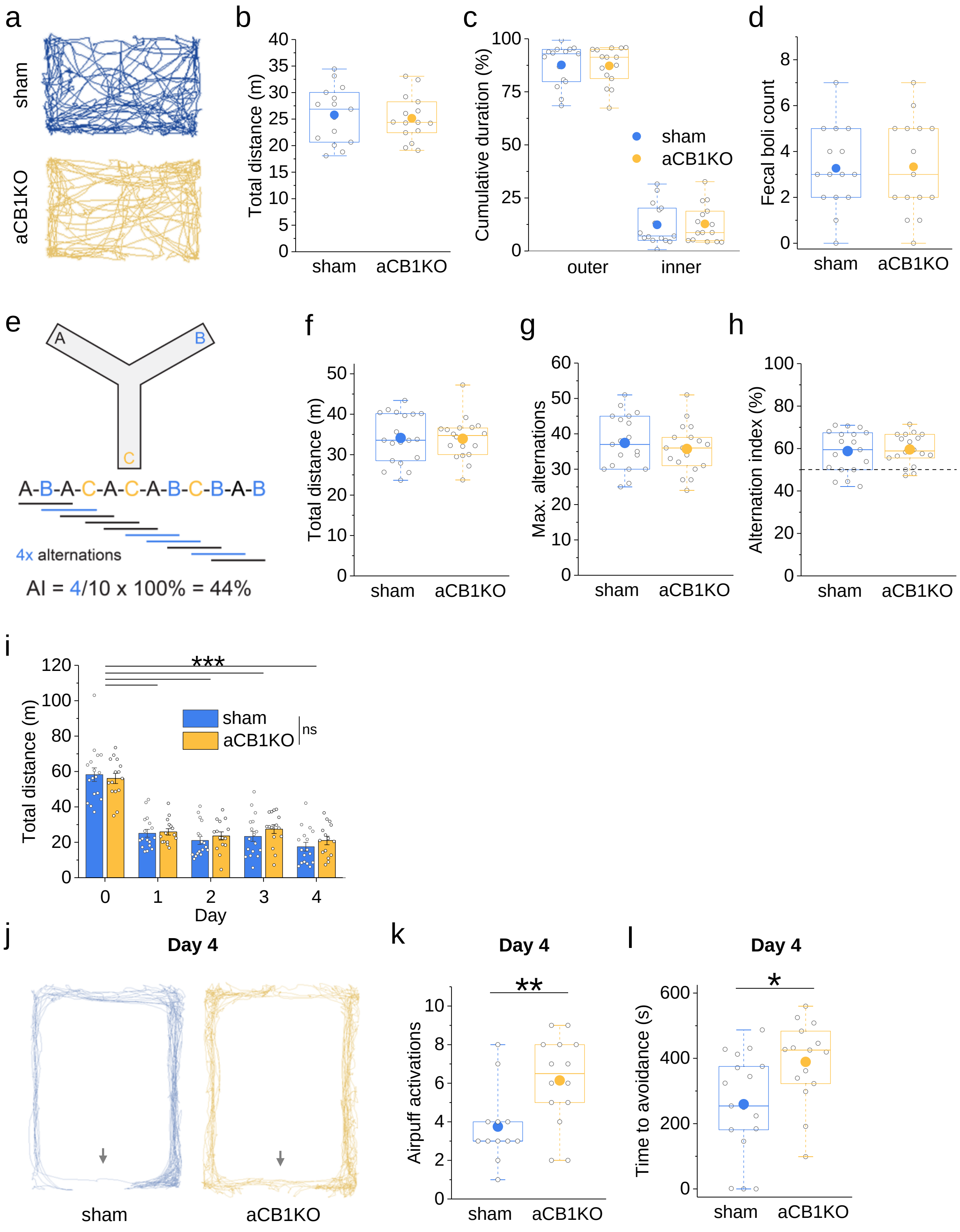

Supplementary figure 8: Absence of astrocytic CB1Rs does not affect the behavior of mice in an open arena or in a spontaneous alternation test (Y-maze). Additional analyses of place avoidance learning in control mice (sham) and mice without astrocytic CB1 receptors (aCB1KO) (Fig. 9). Comparison of GLASTcreERT2 x CBR1 $^{fl/fl}$ x flox-stop tdTomato mice injected with tamoxifen (aCB1KO) or a control solution (sham). For animal model also see **Suppl. Fig. 6-7**.

a) Examples of tracks of mice in an open arena (top: sham, blue; bottom: aCB1KO, yellow).

b-d) The two groups of animals (n = 15 both groups) did not differ significantly regarding the total distance travelled in the arena (**b**, sham: 25.8 ± 1.4 m, aCB1KO: 25.1 ± 1.1 m, t(28) = 0.37, p = 0.71, two-sided Student's t-test), the cumulative time spent in the outer part (less than 8 cm from arena border) or inner part of the arena (**c**, outer, sham: 87. 7 ± 2.5 %, outer, aCB1KO: 87.2 ± 2.3 %, inner, sham: 12.3 ± 2.5 %, inner, aCB1KO 12.8 ± 2.3 %, two-way repeated measurement ANOVA: for groups F(1,28) = 1.00, p = 0.33, for interaction F(1,28) = 0.02, p = 0.90, for inner/outer F(1,28) = 492.49, p < 0.0001) or the number of fecal boli as an indirect measure of anxiety (d, fecal boli count, sham: 3.3 ± 0.5, aCB1KO: 3.3 ± 0.5, t(28) = 0.09, p = 0.93, two-sided Student's t-test).

e) Schematic representation of the spontaneous alternation test (Y-maze) with arms A, B and C. The alternation index (AI) is calculated as the percentage of fully alternating triple entries (blue lines) out of all sequential triplets (blue and black lines).

f-h) The two groups of animals (sham: n = 19, aCB1KO: n = 18) differ not significantly regarding the total distance travelled (**f**, sham: 34.1 ± 1.4 m, aCB1KO: 33.9 ± 1.2 m, t(35) = 0.10, p = 0.92, two-sided Student's t-test), the maximum number of alternations (**g**, sham: 37.4 ± 1.8, aCB1KO: 35.7 ± 1.6, t(35) = 0.71, p = 0.48, two-sided Student's t-test) or the alternation index (**h**, sham: 58.8 ± 2.2, aCB1KO: 59.6 ± 1.7 %, t(35) = 0.29, p = 0.78, two-sided Student's t-test).

i) The total distance travelled by mice declined rapidly during place avoidance learning but did not differ between experimental groups (two-way repeated measures ANOVA; for days F(4,120) = 186.20, p < 0.001, post-hoc Tukey test p < 0.001 as indicated by asterisks; for groups F(1,30) = 0.32, p = 0.58; for interaction F(4,120) = 1.23, p = 0.30; n = 17 and 15 for sham and aCB1KO, respectively).

j) Sample tracks at day 4. The new air puff location is marked by arrows. Note that the sham-treated animals successfully and rapidly avoid the air puff.

k) Number of air puff activations in both groups on day 4. In contrast to **Fig. 9**, animals that did not fully explore the arena on day 3 (no air puff in any location) were excluded from this analysis, because they had no opportunity to notice the absence of an air puff on day 3. This did not change the results qualitatively. Sham-treated animals activated fewer air puffs, i.e., avoided the new air puff area more successfully (sham: 3.75 ± 0.57, n = 12; aCB1KO: 6.14 ± 0.62, n = 14; t(24) = 2.82, p = 0.0096, two-sided Student's t-test).

l) How quickly animals avoided the new air puff location was quantified by measuring the time between the first triggered air puff and the last, after which the air puff area was completely avoided. This time to avoidance is low when mice rapidly acquire the new location of the aversive stimulus. Sham-treated animals displayed a significantly shorter time to avoidance (sham: 259.6 ± 38.2 s, n = 17; aCB1KO: 389.2 ± 32.3 s, n = 15; t(30) = 2.55, p = 0.016, two-sided Student's t-test).

Data are expressed and displayed as mean ± s.e.m. or in box plots. In the latter, the box indicates the 25th and 75th, the whiskers the 5th and 95th percentiles, the horizontal line in the box the median and the mean is represented by a filled circle. Source data are provided as a Source Data file.

Supplementary figure 9

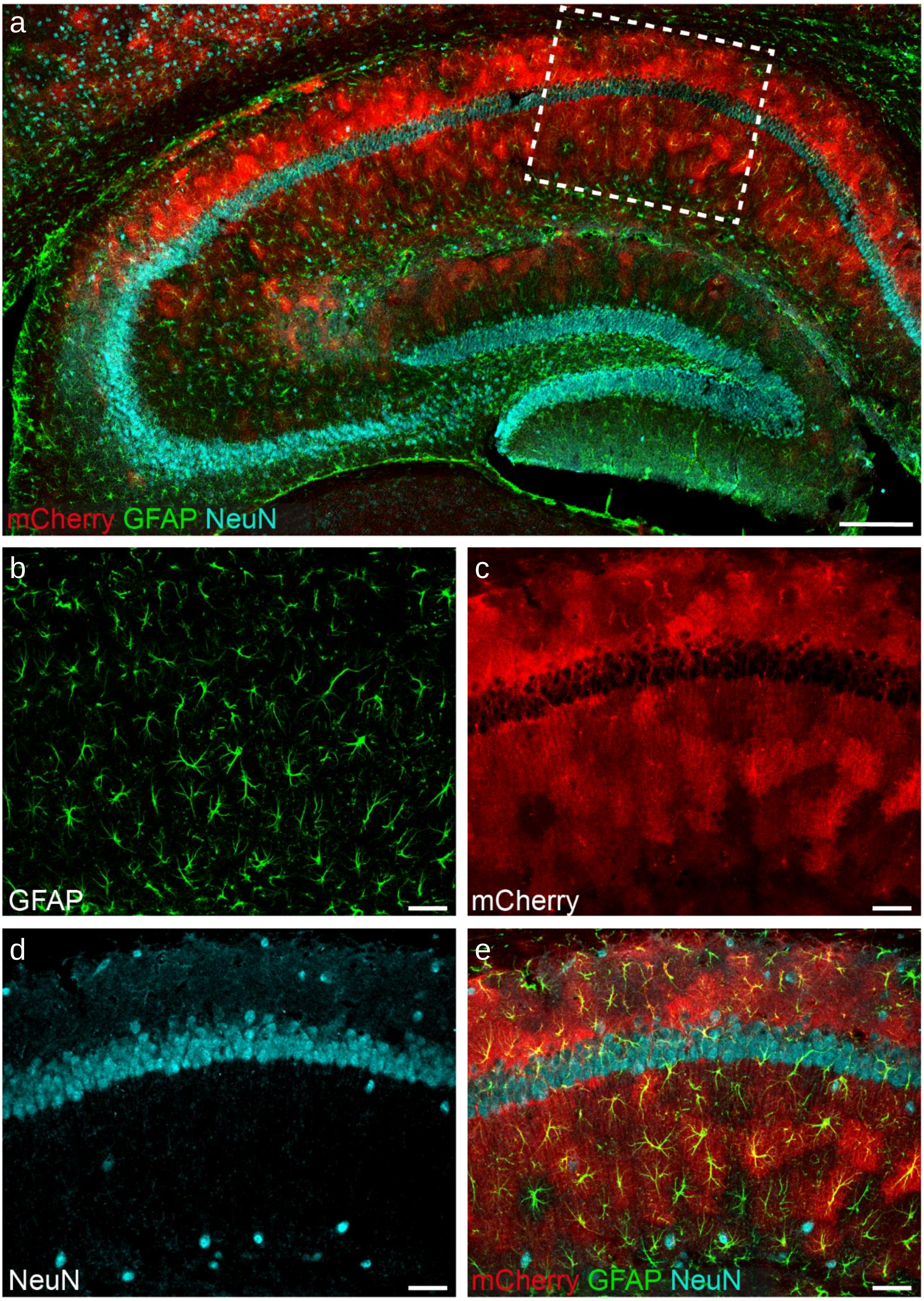

Supplementary figure 9: Characterization of astrocyte-specific expression after stereotactic injection in the CA1 region. C57BL6/N mice were injected bilaterally with AAV.gfaABC1D-mCherry-hPMCA2w/n (CalEx) [5]. Their hippocampal CA1 regions were investigated by immunohistochemistry and confocal microscopy. Scale bars are 200 µm in **a** and 50 µm in **b-e**. Images represent examples from a single experiment.

a) Examples of the hippocampal CA1 region targeted by stereotactic injection. The mCherry expressing cells overlap with astrocytic GFAP expression but not with neuronal NeuN expression. A higher magnification of the delineated area is shown in **b-e**.

b-e) Examples of astrocytic GFAP expression (**b**), mCherry expression showing the typical fluorescence pattern of astrocyte protein expression (**c**), neuronal NeuN expression (**d**) and overlay of GFAP, mCherry and NeuN (**e**).

Supplementary figure 10

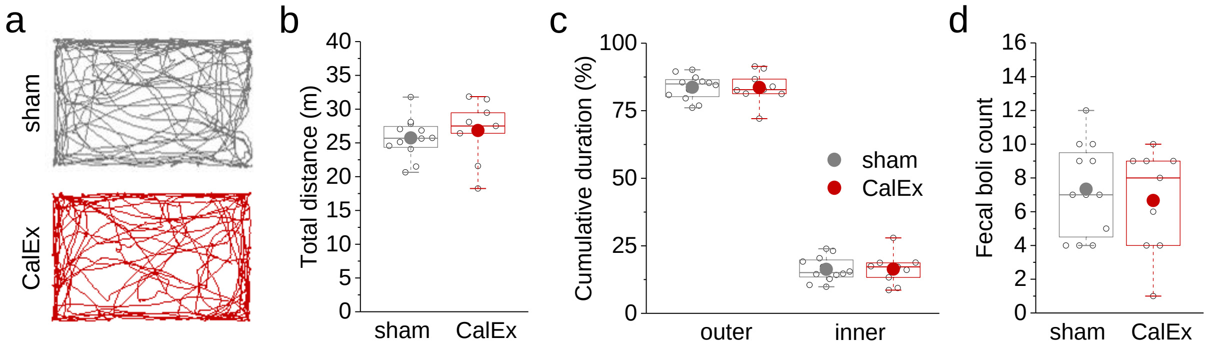

Supplementary figure 10: Expression of the human calcium pump (hPMCA2w/n) in CA1 astrocytes does not affect the movement of mice in an open arena. Comparison of C57BL6/N mice with AAV.gfaABC1D-mCherry-hPMCA2w/n (CalEx) [5] or AAV.gfaABC1D-tdTomato (sham). For animal model also see **Suppl. Fig. 9**.

a) Examples of tracks of mice in an open arena (top: sham, grey; bottom: CalEx, red).

b-d) The two groups of animals (sham = 12; CalEx = 9) did not differ significantly regarding the total distance travelled in the arena (**b**, sham: 25.7 ± 0.86 m, CalEx: 26.8 ± 1.48 m, $t(19) = 0.68$, $p = 0.51$, two-sided Student's t-test), the cumulative time spent in the outer part (less than 8 cm from arena border) or inner part of the arena (**c**, outer, sham: 83.7 ± 1.3 %, outer, CalEx: 83.6 ± 1.9 %, inner, sham: 16.3 ± 1.3 %, inner, CalEx 16.4 ± 1.9 %, two-way repeated measurement ANOVA: for groups $F(1,19) = 0$, $p = 1$, for interaction $F(1,19) = 0.001$, $p = 0.98$, for inner/outer $F(1,19) = 894.80$, $p < 0.0001$) or the number of fecal boli as an indirect measure of anxiety (d, fecal boli count, sham: 7.3 ± 0.8, CalEx: 6.7 ± 1.0, $t(19) = 0.53$, $p = 0.60$, two-sided Student's t-test).

Data are expressed as mean ± s.e.m. in the legend and displayed in box plots. The box indicates the 25th and 75th, the whiskers the 5th and 95th percentiles, the horizontal line in the box the median and the mean is represented by a filled circle. Source data are provided as a Source Data file.

Supplementary figure 11

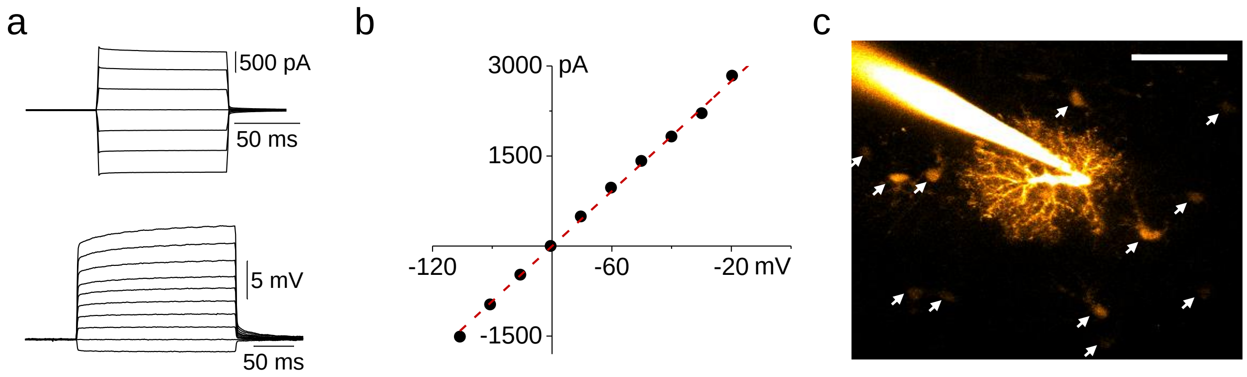

Supplementary figure 11: Identification of cells as astrocyte by whole-cell patch clamp recordings and fluorescence microscopy.

a) Recording from an astrocyte (top: voltage clamp, 10 mV steps; bottom: current clamp, 200 pA steps) displaying the typical ‘passive’ responses of this cell type.

b) Plot of the linear current-voltage relationship of an astrocyte recorded in voltage clamp.

c) Maximum fluorescence intensity projection of gap junction coupled astrocytes filled with Alexa Fluor 594 through the patch pipette (scale bar 50 µm). The image illustrates the typical astrocytic morphology (i.e., highly branched architecture with fine processes) and the cell bodies of neighboring, gap junction coupled astrocytes (white arrows).

Source data are provided as a Source Data file.

Supplementary table 1

parameter	wildtype (mean ± SEM)	sham (mean ± SEM)	aCB1KO (mean ± SEM)	n	p	F value
**Rm (MΩ)**	136.8 ± 14.1	119.3 ± 9.7	132.8 ± 10.5	7/8/9	0.54	F(2,21) = 0.63
**Vm (mV)**	-63.4 ± 2.96	-66.9 ± 1.95	-67.7 ± 1.18	7/8/9	0.21	F(2,21) = 1.70
**AP threshold (mV)**	-43.1 ± 2.11	-50.0 ± 1.88	-48.9 ± 1.09	9/10/10	0.02	F(2,26) = 4.42
**AP amplitude (mV)**	93.1 ± 1.7	93.7 ± 4.0	94.1 ± 1.4	8/10/10	0.97	F(2,25) = 0.03
**AP upstroke (mV/ms)**	409.3 ± 16.8	407.2 ± 9.2	400.1 ± 14.7	9/9/10	0.89	F(2,25) = 0.12

Supplementary table 1. Properties of CA1 pyramidal cells in wildtype, sham-injected mice and mice with deletion of astrocytic CB1 receptors (aCB1KO). Also see Supplementary figures 6 and 7). The following parameters were analyzed in whole-cell patch clamp and field recordings: the membrane resistance (R_M), the resting membrane potential (V_M), the action potential (AP) threshold, AP amplitude and AP maximum depolarization rate (upstroke) in alveus stimulation experiments and compared using one-way ANOVA. Post-hoc analysis for AP threshold (Tukey): q = 3.95, p = 0.025 for wildtype vs. sham, q = 3.31, p = 0.068 for wildtype vs. aCB1KO, q = 0.65, p = 0.89 for sham vs. aCB1KO. Data are expressed as mean ± s.e.m. Source data are provided as a Source Data file.

Supplementary table 2

Figure	parameter		n	|test|	p value
**1d**	threshold stimulus		16	t(15) = 5.37	0.000077
	slow component		16	t(15) = 8.91	0.00000022
**1e**	threshold stimulus		16	t(15) = 5.21	0.00011
	slow component		14	t(13) = 5.00	0.00024
**2d**	threshold		6	t(5) = 2.78	0.039
	slow component		6	t(5) = 4.07	0.0097
**2e**	threshold		6	t(5) = 2.59	0.049
	slow component		6	t(5) = 2.65	0.045
**3b**	R/R_0	control	13	t(12) = 3.38	0.0054
		in AM251	5	t(4) = 0.71	0.52
**3c**	threshold stimulus		9	t(8) = 3.24	0.012
	slow component		8	t(7) = 2.62	0.034
**3d**	threshold stimulus		5	t(4) = 0.16	0.88
	slow component		5	t(4) = 1.31	0.26
**3e**	threshold stimulus		6	t(5) = 0.17	0.87
	slow component		6	t(5) = 0.50	0.64
**3f**	threshold stimulus		6	t(5) = 0.27	0.80
	slow component		6	t(5) = 0.21	0.84
**4c**	R/R_0 relative to baseline		9	t(8) = 3.04	0.016
**4d** (S3a)	threshold stimulus	4 Hz	5	t(4) = 0.00	0.99
		10 Hz & 40 Hz	8	F(2,14) = 17.4	0.00016
		post-hoc 10 Hz		t(14) = 5.56	0.000071
		post-hoc 40 Hz		t(14) = 1.05	0.31
		post-hoc 10 Hz vs. 40 Hz		t(14) = 4.50	0.00050
		20 Hz & 5 min after (S3a)	9	$\chi^2(2) = 9.06$	0.011
		post-hoc 20 Hz		z = 2.12	0.031
		post-hoc 5 min		z = 1.26	0.21
		post-hoc 20 Hz vs. 5 min		z = 2.61	0.0039
	change	4 Hz, 10 Hz, 20 Hz & 40 Hz	5/8/9/8	F(3,26) = 5.51	0.0046
		post-hoc 4 Hz vs. 10 Hz		t = 3.67	0.0011
		post-hoc 10 Hz vs. 20 Hz		t = 1.95	0.049
		post-hoc 10 Hz vs. 40 Hz		t = 3.17	0.0039
**4e** (S3b)	slow component	4 Hz	5	t(4) = 1.40	0.23
		10 Hz & 40 Hz	8	F(2,14) = 19.30	0.000095
		post-hoc 10 Hz		t(14) = 5.75	0.000051
		post-hoc 40 Hz		t(14) = 0.84	0.41
		post-hoc 10 Hz vs. 40 Hz		t(14) = 4.91	0.00069
		20 Hz & 5 min after (S3b)	8	F(2,14) = 5.80	0.015
		post-hoc 20 Hz		t(14) = 2.57	0.022
		post-hoc 5 min		t(14) = 0.66	0.52
		post-hoc 20 Hz vs. 5 min		t(14) = 3.22	0.0061
	change	4 Hz, 10 Hz, 20 Hz & 40 Hz	5/8/8/8	$\chi^2(3) = 8.77$	0.032
		post-hoc 4 Hz vs. 10 Hz		z = 1.98	0.045
		post-hoc 10 Hz vs. 40 Hz		z = 2.36	0.015
**4f**	threshold stimulus		7	t(6) = 0.66	0.53
	slow component		7	t(6) = 0.20	0.85
**4g**	threshold stimulus		8	t(7) = 0.35	0.73
	slow component		8	t(7) = 1.20	0.27

Figure	parameter				n	|test|	p value
**5c**	ΔR/R_0 relative to baseline				26/25	z = 4.31	0.0000046
**5d**	threshold stimulus			10 Hz in ZD7288	8	t(7) = 1.05	0.33
				WIN55 in ZD7288	10	t(9) = 2.68	0.025
	change			10 Hz control, 10 Hz in ZD7288 & WIN55 in ZD7288	8/8/10	$\chi^2(2)$ = 14.06	0.00088
				post-hoc 10 Hz control vs. 10 Hz in ZD7288		z = 3.10	0.00062
				post-hoc 10 Hz control vs. WIN55 in ZD7288		z = 1.02	0.32
				post-hoc 10 Hz vs. WIN55 in ZD7288		z = 3.15	0.00055
**5e**	slow component			10 Hz in ZD7288	8	t(7) = 0.67	0.52
				WIN55 in ZD7288	10	t(9) = 4.77	0.0010
	change			10 Hz control, 10 Hz in ZD7288 & WIN55 in ZD7288	8/8/10	$\chi^2(2)$ = 10.53	0.0052
				post-hoc 10 Hz control vs. 10 Hz in ZD7288		z = 2.68	0.0047
				post-hoc 10 Hz control vs. WIN55 in ZD7288		z = 0.49	0.63
				post-hoc 10 Hz vs. WIN55 in ZD7288		z = 2.80	0.0031
**5g**	change			control vs. ZD7288	8/6	z = 3.04	0.000666
				control vs. 0	8	z = 2.45	0.00781
				ZD7288 vs. 0	6	z = 1.62	0.125
**6c**	threshold stimulus			WT	7	t(6) = 4.94	0.0026
				sham	8	t(7) = 5.74	0.00071
				aCB1KO	9	z = 0	1
				D-serine in aCB1KO	7	t(6) = 9.16	0.000096
	change			WT, sham & aCB1KO	7/8/9/7	F(3,27) = 9.94	0.00014
				post-hoc WT vs. sham		0.45	0.66
				post-hoc WT vs. aCB1KO		4.67	0.000072
				post-hoc sham vs. aCB1KO		4.370	0.00016
				post-hoc aCB1KO vs. D-serine in aCB1KO		3.84	0.00067
**6d**	slow component			WT	7	t(6) = 2.80	0.031
				sham	8	t(7) = 3.95	0.0056
				aCB1KO	9	t(8) = 1.66	0.14
				D-serine in aCB1KO	7	T(6) = 5.81	0.0011
	change			WT, sham & aCB1KO	7/8/9/7	F(3,27) = 7.61	0.00076
				post-hoc WT vs. sham		t = 0.85	0.40
				post-hoc WT vs. aCB1KO		t = 3.08	0.0047
				post-hoc sham vs. aCB1KO		t = 4.10	0.00034
				post-hoc aCB1KO vs. D-serine in aCB1KO		t = 3.95	0.00050
**8b**	total exploration				10/12	z = 0.63	0.54
**8c**	discrimination index				10/12	t(20) = 2.86	0.0098
**9b**	transition position A			day 1	17/15	z = 0.50	0.62
				day 2	17/15	z = 0.36	0.73
				day 3	17/15	z = 0.76	0.45
		inset	day 1	time	17/15	F(1,30) = 139.71	< 0.00001
				treatment		F(1,30) = 0.22	0.64
			day 3	time	17/15	F(1,30) = 16.60	0.00031
				treatment		F(1,30) = 0.36	0.55
				interaction		F(1,30) = 0.43	0.52
**9c**	transitions			position A	17/15	z = 0.29	0.77
				position B	17/15	t(30) = 2.92	0.0066
		inset		time	17/15	F(1,30) = 28.42	0.0000092
				treatment		F(1,30) = 8.51	0.0066
				interaction		F(1,30) = 3.48	0.072
				post-hoc 1st time bin		t(30) = 5.04	0.0066
				post-hoc 2nd time bin		t(30) = 1.68	0.64
				post-hoc sham		t(30) = 3.21	0.13
				post-hoc aCB1KO		t(30) = 6.27	0.00063

Figure	parameter		n	|test|	p value
**10b**	threshold stimulus	sham	6	t(5) = 5.85	0.0021
		CalEx	5	t(4) = 0.0008	1.00
	change	sham vs. CalEx	6/5	t(9) = 4.74	0.0011
**10c**	slow component	sham	6	t(6) = 3.56	0.016
		CalEx	5	t(4) = 1.43	0.23
	change	sham vs. CalEx	6/5	t(9) = 3.44	0.0073
**10e**	total exploration		7/5	t(10) = 0.38	0.71
**10f**	discrimination index		7/5	t(10) = 2.81	0.019

Supplementary table 2: Detailed information on statistics. Please see figure legends for the used tests.

References

1. Gee, J. M. *et al.* Imaging Activity in Neurons and Glia with a Polr2a-Based and Cre-Dependent GCaMP5G-IRES-tdTomato Reporter Mouse. *Neuron* **83**, 1058–1072 (2014).
2. Wang, Y. *et al.* Accurate quantification of astrocyte and neurotransmitter fluorescence dynamics for single-cell and population-level physiology. *Nat. Neurosci.* **22**, 1936–1944 (2019).
3. Mori, T. *et al.* Inducible gene deletion in astroglia and radial glia—A valuable tool for functional and lineage analysis. *Glia* **54**, 21–34 (2006).
4. Madisen, L. *et al.* A robust and high-throughput Cre reporting and characterization system for the whole mouse brain. *Nat Neurosci* **13**, 133–140 (2010).
5. Yu, X. *et al.* Reducing Astrocyte Calcium Signaling In Vivo Alters Striatal Microcircuits and Causes Repetitive Behavior. *Neuron* **99**, 1170–1187 (2018).
