# Supplementary material for: An astrocytic signaling loop for frequency-dependent control of dendritic integration and spatial learning
Source: Nat Commun. 2022 Dec 24;13:7932. doi: 10.1038/s41467-022-35620-8 (PMC9789958; doi:10.1038/s41467-022-35620-8)
Supplement: Supplementary file 3 — Reporting Summary [file 41467_2022_35620_MOESM3_ESM.pdf]

nature portfolio

Corresponding author(s): Christian Henneberger
Last updated by author(s): Nov 25, 2022

Reporting Summary

Nature Portfolio wishes to improve the reproducibility of the work that we publish. This form provides structure for consistency and transparency in reporting. For further information on Nature Portfolio policies, see our Editorial Policies and the Editorial Policy Checklist.

Statistics

For all statistical analyses, confirm that the following items are present in the figure legend, table legend, main text, or Methods section.

n/a	Confirmed	
☐	☒	The exact sample size (*n*) for each experimental group/condition, given as a discrete number and unit of measurement
☐	☒	A statement on whether measurements were taken from distinct samples or whether the same sample was measured repeatedly
☐	☒	The statistical test(s) used AND whether they are one- or two-sided<br>*Only common tests should be described solely by name; describe more complex techniques in the Methods section.*
☒	☐	A description of all covariates tested
☐	☒	A description of any assumptions or corrections, such as tests of normality and adjustment for multiple comparisons
☐	☒	A full description of the statistical parameters including central tendency (e.g. means) or other basic estimates (e.g. regression coefficient) AND variation (e.g. standard deviation) or associated estimates of uncertainty (e.g. confidence intervals)
☐	☒	For null hypothesis testing, the test statistic (e.g. *F*, *t*, *r*) with confidence intervals, effect sizes, degrees of freedom and *P* value noted<br>*Give P values as exact values whenever suitable.*
☒	☐	For Bayesian analysis, information on the choice of priors and Markov chain Monte Carlo settings
☒	☐	For hierarchical and complex designs, identification of the appropriate level for tests and full reporting of outcomes
☒	☐	Estimates of effect sizes (e.g. Cohen's *d*, Pearson's *r*), indicating how they were calculated

Our web collection on statistics for biologists contains articles on many of the points above.

Software and code

Policy information about availability of computer code

Data collection	Commercial software: pClamp 10.3, FluoView 4.2, ScanImage 3.8. No custom software/code was used.
Data analysis	Commercial and open source software: pClamp 10.3, Igor PRO 9.0, Matlab 2020, OriginPro 2017, EthoVision XT 14.0, FIJI 2.3.0. No custom software/code was used.

For manuscripts utilizing custom algorithms or software that are central to the research but not yet described in published literature, software must be made available to editors and reviewers. We strongly encourage code deposition in a community repository (e.g. GitHub). See the Nature Portfolio guidelines for submitting code & software for further information.

Data

Policy information about availability of data

All manuscripts must include a data availability statement. This statement should provide the following information, where applicable:

- Accession codes, unique identifiers, or web links for publicly available datasets
- A description of any restrictions on data availability
- For clinical datasets or third party data, please ensure that the statement adheres to our policy

The complete data set generated in this study is described and provided in this document, in the Supplementary Information and the Source Data file. Source data are provided with this paper. Further raw data supporting the current study have not been deposited in a public repository because of their highly diverse nature and formats. They are available from the lead contact upon request.

Human research participants

Policy information about studies involving human research participants and Sex and Gender in Research.

Reporting on sex and gender	N/A
Population characteristics	N/A
Recruitment	N/A
Ethics oversight	N/A

Note that full information on the approval of the study protocol must also be provided in the manuscript.

Field-specific reporting

Please select the one below that is the best fit for your research. If you are not sure, read the appropriate sections before making your selection.

☒ Life sciences ☐ Behavioural & social sciences ☐ Ecological, evolutionary & environmental sciences

For a reference copy of the document with all sections, see nature.com/documents/nr-reporting-summary-flat.pdf

Life sciences study design

All studies must disclose on these points even when the disclosure is negative.

Sample size

Effect sizes could not be estimated before the experiment because similar data was not available. Therefore, a power analysis and an estimation of the required sample size could not be performed before the experiment. Therefore, we have used sample sizes that are customary in this research field. See for instance:

Müller C et al. (2012). Inhibitory Control of Linear and Supralinear Dendritic Excitation in CA1 Pyramidal Neurons. Neuron 75, 851–864.

Robin LM et al. (2018). Astroglial CB1 Receptors Determine Synaptic D-Serine Availability to Enable Recognition Memory. Neuron 98, 935–944.

Data exclusions

There were several exclusion criteria depending on the experiment. The criteria are fully stated in the manuscript whenever data was excluded. They were:

1) Whole-cell patch clamp: Recordings in which the access resistance exceeded 30 MΩ were excluded from the analysis.

2) Ca2+ signal analysis, AQuA: Somatic events, events with a duration of less than 3 frames and events with a negative decay time constant were excluded.

3) Behavioural tests, object location test: Animals that showed excessive climbing or little exploration (exploration time <5 s in 10 min or <2 s in 5 min; total distance <1 m in 5 min) as well as outliers for the analyzed parameters (mean ± 2SD) were excluded (tamoxifen injected mice: n = 5 out of 37 mice; virus injected mice: n = 9 out of 21 mice).

4) Behavioural tests, Y-maze: Animals were considered to have an intrinsic arm preference or aversion when they entered an arm 10 % more or less often than expected (33%, relative arm visits < 23% or > 43%). Those animals were excluded from further analysis (n = 3).

Replication

All attempts at replication were successful. The number of replicates and independent experiments is given throughout the paper and indicated by 'n'.

Randomization

Animals, slices and cells were pseudo-randomly assigned to experimental groups.

Blinding

Blinding was done for all behavioural test. No blinding was done in other experiments, because the same persons did all experimental procedures and analyses and could therefore identify the experimental group/condition during analysis.

Behavioural & social sciences study design

All studies must disclose on these points even when the disclosure is negative.

Study description

Briefly describe the study type including whether data are quantitative, qualitative, or mixed-methods (e.g. qualitative cross-sectional, quantitative experimental, mixed-methods case study).

Research sample	*State the research sample (e.g. Harvard university undergraduates, villagers in rural India) and provide relevant demographic information (e.g. age, sex) and indicate whether the sample is representative. Provide a rationale for the study sample chosen. For studies involving existing datasets, please describe the dataset and source.*
Sampling strategy	*Describe the sampling procedure (e.g. random, snowball, stratified, convenience). Describe the statistical methods that were used to predetermine sample size OR if no sample-size calculation was performed, describe how sample sizes were chosen and provide a rationale for why these sample sizes are sufficient. For qualitative data, please indicate whether data saturation was considered, and what criteria were used to decide that no further sampling was needed.*
Data collection	*Provide details about the data collection procedure, including the instruments or devices used to record the data (e.g. pen and paper, computer, eye tracker, video or audio equipment) whether anyone was present besides the participant(s) and the researcher, and whether the researcher was blind to experimental condition and/or the study hypothesis during data collection.*
Timing	*Indicate the start and stop dates of data collection. If there is a gap between collection periods, state the dates for each sample cohort.*
Data exclusions	*If no data were excluded from the analyses, state so OR if data were excluded, provide the exact number of exclusions and the rationale behind them, indicating whether exclusion criteria were pre-established.*
Non-participation	*State how many participants dropped out/declined participation and the reason(s) given OR provide response rate OR state that no participants dropped out/declined participation.*
Randomization	*If participants were not allocated into experimental groups, state so OR describe how participants were allocated to groups, and if allocation was not random, describe how covariates were controlled.*

Ecological, evolutionary & environmental sciences study design

All studies must disclose on these points even when the disclosure is negative.

Study description	*Briefly describe the study. For quantitative data include treatment factors and interactions, design structure (e.g. factorial, nested, hierarchical), nature and number of experimental units and replicates.*
Research sample	*Describe the research sample (e.g. a group of tagged Passer domesticus, all Stenocereus thurberi within Organ Pipe Cactus National Monument), and provide a rationale for the sample choice. When relevant, describe the organism taxa, source, sex, age range and any manipulations. State what population the sample is meant to represent when applicable. For studies involving existing datasets, describe the data and its source.*
Sampling strategy	*Note the sampling procedure. Describe the statistical methods that were used to predetermine sample size OR if no sample-size calculation was performed, describe how sample sizes were chosen and provide a rationale for why these sample sizes are sufficient.*
Data collection	*Describe the data collection procedure, including who recorded the data and how.*
Timing and spatial scale	*Indicate the start and stop dates of data collection, noting the frequency and periodicity of sampling and providing a rationale for these choices. If there is a gap between collection periods, state the dates for each sample cohort. Specify the spatial scale from which the data are taken*
Data exclusions	*If no data were excluded from the analyses, state so OR if data were excluded, describe the exclusions and the rationale behind them, indicating whether exclusion criteria were pre-established.*
Reproducibility	*Describe the measures taken to verify the reproducibility of experimental findings. For each experiment, note whether any attempts to repeat the experiment failed OR state that all attempts to repeat the experiment were successful.*
Randomization	*Describe how samples/organisms/participants were allocated into groups. If allocation was not random, describe how covariates were controlled. If this is not relevant to your study, explain why.*
Blinding	*Describe the extent of blinding used during data acquisition and analysis. If blinding was not possible, describe why OR explain why blinding was not relevant to your study.*

Did the study involve field work? ☐ Yes ☒ No

Reporting for specific materials, systems and methods

We require information from authors about some types of materials, experimental systems and methods used in many studies. Here, indicate whether each material, system or method listed is relevant to your study. If you are not sure if a list item applies to your research, read the appropriate section before selecting a response.

Materials & experimental systems

n/a	Involved in the study
	[x] Antibodies
[x]	Eukaryotic cell lines
[x]	Palaeontology and archaeology
	[x] Animals and other organisms
[x]	Clinical data
[x]	Dual use research of concern

Methods

n/a	Involved in the study
[x]	ChIP-seq
[x]	Flow cytometry
[x]	MRI-based neuroimaging

Antibodies

Antibodies used	GFAP (polyclonal rabbit anti-cow GFAP, 1:500; Z0334, Dako; polyclonal chicken anti-human GFAP, 1:500, 173 006, Synaptic Systems), NeuN (monoclonal mouse anti NeuN, 1:200; clone A60, MAB377, Chemicon), anti-rabbit (polyclonal goat anti-rabbit IgG (H+L)-Alexa Fluor 488, 1:500; A11034, Ivitrogen), anti-mouse (polyclonal goat anti-mouse IgG (H+L)-biotin, 1:500; 115-065-003, Dianoca), anti-chicken (polyclonal goat anti-chicken IgG (H+L)-Alexa Fluor 488, 1:500; A11039, Invitrogen)
Validation	anti-GFAP Z0334, validation by supplier : not available<br><br>anti-GFAP 173 006, validation by supplier: reacts with human (P14136), rat (P47819), mouse (P03995), specific for GFAP, detects all isoforms. K.O.<br><br>anti-NeuN, MAB377: validation by supplier, specificity: "... reacts with most neuronal cell types throughout the nervous system of mice including cerebellum, cerebral cortex, hippocampus, thalamus, spinal cord and neurons in the peripheral nervous system including dorsal root ganglia, sympathetic chain ganglia and enteric ganglia. Developmentally, immunoreactivity is first observed shortly after neurons have become postmitotic, no staining has been observed in proliferative zones. The immunohistochemical staining is primarily localized in the nucleus of the neurons with lighter staining in the cytoplasm. The few cell types not reactive with MAB377 include Purkinje, mitral and photoreceptor cells."

Animals and other research organisms

Policy information about studies involving animals; ARRIVE guidelines recommended for reporting animal research, and Sex and Gender in Research

Laboratory animals	Animal strains and lines: Wistar rats (Charles River), C57BL/6N wild type mice (Charles River), transgenic mice of the following lines: GLAST-creERT2 (Mori et al. 2006), CB1fl/fl (Marsicano et al., 2003), flox-top tdTomato (Madisen et al. 2010) (JAX 007908), flox-stop GCaMP5g-IRES-tdTomato. All transgenic mouse lines were bred to have an C57BL/6N background.<br><br>Age: Rats were 3-5 weeks old. All mice were 6-10 weeks old.<br><br>Housing: All animals were housed under 12 h light/dark conditions and were allowed ad libitum access to food and water (room temperature 22 °C, air humidity 50-70 %).
Wild animals	No wild animals were used in the study.
Reporting on sex	Sex-based analyses were not performed. Sex of animals is clearly stated in the methods section of the manuscript.
Field-collected samples	No field collected samples were used in the study.
Ethics oversight	HET of the University of Bonn, LANUV of the regional government of Northrhine-Westphalia, Germany

Note that full information on the approval of the study protocol must also be provided in the manuscript.
